# The Persistence of Self-Provisioning Among Smallholder Farmers in Northeast Madagascar

**DOI:** 10.1007/s10745-015-9791-8

**Published:** 2015-11-24

**Authors:** Rheyna Laney, B. L. Turner

**Affiliations:** Department of Geography and Global Studies, Sonoma State University, Rohnert Park, CA USA; School of Geographical Sciences and Urban Planning & School of Sustainability, Arizona State University, Tempe, USA

**Keywords:** Dual production systems, Swidden, Subsistence, Self-provisioning, Food security, Commercialization, Induced intensification, Agricultural change, Madagascar, Africa

## Abstract

In the Andapa region of northeast Madagascar, smallholders cultivating swidden hill rice (*tavy*) for subsistence are pressing against neighboring nature reserves. A dominant policy approach to reducing this pressure requires that smallholders abandon *tavy* and purchase rice from proceeds obtained from their environmentally sustainable commercial crops, vanilla and coffee. Economic liberalization policies have succeeded in stimulating the expansion of these commercial crops, but have failed to reduce *tavy* production. We ask why this dual (subsistence and commercial) production system persists. We test two explanatory views: that either market imperfections deny farmers full entry into the market, or that internal production goals or socio-cultural norms create barriers to full market participation. Results support the latter view, although not for reasons that have been associated with this view in past studies. We propose a new factor that may serve as a barrier to full-market immersion among Andapa *tavy* farmers, the social relations of property.

## Introduction

**Fig. 1:**
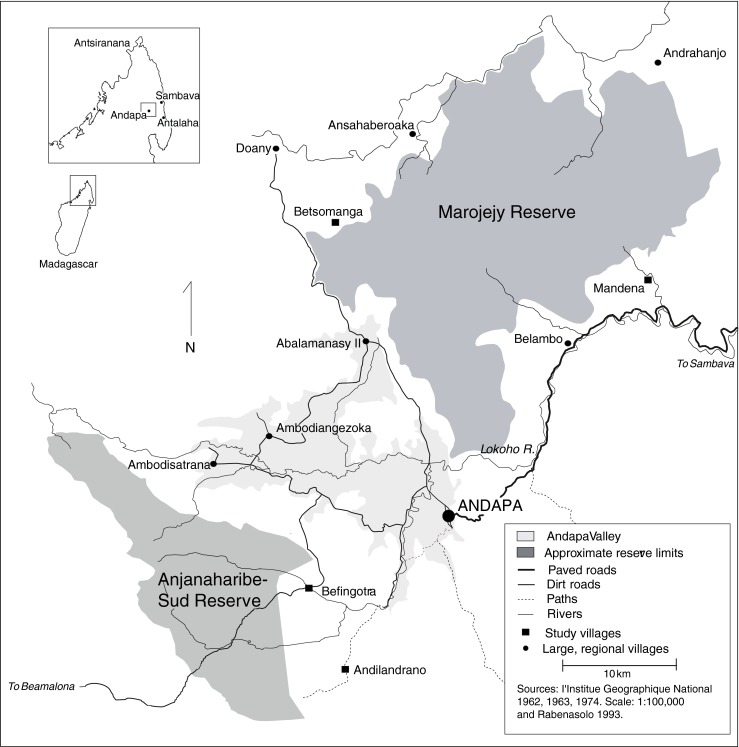
The Andapa region

Smallholder farmers in the Andapa Region of northeast Madagascar (Fig. 1) practice a dual production system involving slash-and-burn hill rice (*tavy*) and irrigated paddy rice for subsistence and vanilla and coffee for the market. Population growth has triggered land pressure, spurring the search for new land for cultivation inside the region’s two nature reserves. The dominant approach to reducing pressure on these reserves and others throughout eastern Madagascar proposes redirecting surrounding communities into more intensive, reliable, and sustainable pathways of agricultural development (Erdmann 2003; Kistler and Spack 2003; Messerli 2000; WWF-Madagascar 2012).

Where irrigable land exists, farmers are encouraged to shift from *tavy* to paddy – a pathway that does not require giving up subsistence production (Messerli 2000; IFAD 2012). Where irrigable land is insufficient, as in the foothills surrounding the Andapa Valley, farmers either will have to adopt more labor-intensive practices within the *tavy* sector (Styger *et al.*^2007^) or abandon self-provisioning and purchase rice from the proceeds of coffee and vanilla sales. Planted compactly, the permanent rooting of these cash crops binds the soil, permitting the viable and sustained use of steep slopes.

Madagascar liberalized its markets in the late 1980s to the early 1990s, tacitly supporting the cash-crop pathway. Some Andapa farmers responded by enlarging their commercial fields. None, however, enacted subsistence substitution, giving up rice production to purchase this staple with profits from vanilla and coffee crops. *Tavy* still dominates the landscape.

This case invokes the long-standing, unresolved question of why many smallholders around the world maintain self-provisioning rather than integrate fully into the market economy (Netting 1974; Goodman and Redclift 1982; Van der Ploeg 2008; Schmook *et al.*^2013^). The problem may be framed by two broad perspectives, labeled here as exogenous and endogenous views. The exogenous view holds that smallholders are “penny capitalists,” constrained from commercializing fully by market imperfections or capitalist institutions of exploitation (Vogt 1969; Goodman and Redclift 1982; Dorward *et al.*^1998^). The endogenous view maintains subsistence production logics or cultural norms contextualizing smallholder economies impede full-market immersion (Hyden 1980; Turner and Brush 1987; Turner and Ali 1996).

This study explores why *tavy* persists in Andapa from these two perspectives. We conclude that a cultural norm ensuring subsistence security is at work, but find the explanations commonly associated with this endogenous thesis irrelevant to the Andapa case. We hypothesize that part of the explanation may reside in the social relations of property (Ferguson 1985, 1990), with implications for current development policies.

## The Persistence of Subsistence-Market (Dual) Production Systems

The exogenous view embodies two different interpretations about the constraints impeding full-market immersion. Neoclassic economists assert that smallholders are entrepreneurs-in-waiting, denied full access to markets by imperfect conditions. Under favorable conditions farmers engage the market, raising aspirations, and ultimately move to market optimizing behavior (Schultz 1964; Popkin 1980; Mellor and Desai 1985). Impediments to that transformation are attributed to market failures, such as inadequate input and credit markets, or factors that cause high transaction costs, such as inadequate transportation (Dorward *et al.*^1998^; Kherallah and Kirsten 2001). Political economists, in contrast, argue that capitalist systems, especially as practiced historically in Africa, seek to maintain dual production systems in order to obtain a cheap supply of farm goods by having farmers feed themselves (Bernstein 1977; Goodman and Redclift 1982; Peet 1991). Together, these arguments place the explanation for self-provisioning on factors external to the smallholder. The political economy interpretation is not tested in this study as we have no means to address it empirically.

The endogenous view highlights factors embedded within smallholders themselves that make a shift to full-market production a difficult or unwanted transition. One set of interpretations holds that smallholders maintain a production logic that emphasizes drudgery of labor (e.g., Chayanov 1966; Barlett 1976), avoidance of risk to base consumption needs (e.g., Lipton 1968), or aspirations other than profit-maximization (e.g., Brookfield 1984). As such, the intensification of subsistence production is induced by increasing demand (e.g., population growth) in the face of land constraints (Boserup 1965; Turner and Brush 1987). This process may push subsistence farmers into dual systems (or even full-market immersion) when commercial production meets consumption goals more efficiently than subsistence (Boserup 1981; Tiffen and Mortimore 1992).

Another set of interpretations holds that subsistence is enmeshed in a group behavior aimed at protecting internal values and socio-cultural norms that preserve autonomy (e.g.*,* Van der Ploeg 2008), equity (e.g.*,* Wolf 1957; Foster 1965), rights to minimum subsistence (e.g.*,* Scott 1976; Hyden 1980), or socio-religious systems (e.g.*,* Rappaport 1984). In these cases, the move to full-market cropping requires changes in these values, norms, or institutions. Missing in this version is the role that social relations of property (Ferguson 1985, 1990) may play in securing subsistence.

## The Andapa Context

### The Political Economy of Commercial Production

In the late 1800s, French and Reunionese colonial settlers established coffee and vanilla plantations in the sparsely-populated Andapa Valley, drawing in Malagasy immigrant labor dominated by the Tsimihety ethnic group (Fig. 1) (Neuvy 1989). These workers soon quit, claimed land in the surrounding foothills, and adopted the dual production system that remains today (Cabanes 1982). By 1925, these smallholders produced 90 % of Andapa’s vanilla, forming a corner of the “vanilla triangle” (Fig. 1: inset) that soon became the largest producer of high-quality bourbon vanilla in the world (Portais 1972). Middlemen bought, processed and sold the commercial crops to French trading companies (Althabe 1968). Smallholders in the irrigable Andapa Valley proper (Fig. 1) eventually shifted their commercial sector into paddy, becoming a “rice bowl” for northeast Madagascar. Those in the surrounding foothills (the subject of this study) maintained coffee and vanilla production (Neuvy 1989).

Madagascar’s post-colonial socialist drift in the 1970s witnessed strong commodity regulations. The state set exceptionally low rice prices to subsidize its urban population, but black markets forced deregulation by the mid-1980s (Pryor 1990). The state controlled the coffee and vanilla sectors through a marketing board, CAVAGI (Caisse de Commercialisation et de Stablilisation des Prix du Café, de la Vanille et du Girofle), which set relatively low, but steady, farm-gate prices (Krivonos 2004). The state also colluded with Comoros and Réunion to create a vanilla cartel that kept exports low and international prices exceptionally high, garnering high taxes (Melo *et al.*^2000^).

**Fig. 2:**
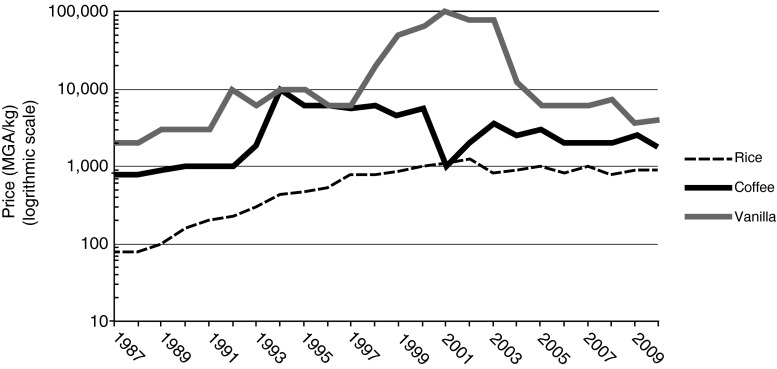
Andapa Farm-Gate Prices. Average price within a season. Sources: 1987–2003: Ramandriabe Ltd., Andapa and DUBOSC R & Cie., Andapa; 2004–2010: Key informant: Pascaline Lahady Charlotte; MGA = Malagasy Ariary; 1 USD = ~1100 MGA in 1987; 5000 MGA in 1997; ~1800 MGA in 2007

During a mid-1980s fiscal crisis, the IMF pressured Madagascar to liberalize its economy, forcing CAVAGI to release its control of coffee by the late 1980s and vanilla by 1994 (Pryor 1990; Krivonos 2004). Coffee farm-gate prices did not change significantly until global prices rose in the mid-1990s and collapsed again in 2001–02 (Fig. 2). Vanilla prices initially rose, but without CAVAGI to hold down supply, they soon fell again (Cadot *et al.*^2009^).

In the early 2000s, political unrest and destructive cyclones in Indonesia and Madagascar reduced global supply, and prices reached nearly $500/kg USD. Global competitors quickly entered the market and consumers switched to cheaper, artificial vanillin, causing prices to plunge again (Fig. 2).

### Land Management

The earliest Tsimihety immigrants established the land tenure system that endures today. Farmers cleared plots on the lower reaches of a watershed and claimed ownership of the upper reaches as well, thereby establishing large landholdings with the most irrigable land for paddy. Subsequent immigrants had to confirm that no prior claims encumbered a particular watershed before establishing their claim. Newer immigrants have had to purchase land.

Land resources are organized in a mixed private and extended-family system. *Tavy* and paddy fields are usually owned and managed at the family level, often embracing multiple households spanning two or three generations. Family elders temporarily allocate fields to individual households for *tavy* or paddy, but grant parcels as private property to adults (males and females ~16 years of age) for commercial crops. In this way, most farmers have full ownership over a small amount of land and contingent access to more, with families operating as the encompassing land management unit. When elders die, families divide the *tavy* and paddy land amongst the next generation, both males and females. If the recipient already has adult children, that subsection of land continues to be managed by an extended family. Single-household recipients manage their land as private property until their own children come of age, at which point they transform into multiple-household families again.

*Tavy* is managed in a short-fallow system (typically 1:3 to 1:7 crop to fallow years) with minimal attention during fallows (Laney 2002). Paddy fields are cultivated twice a year where water is sufficient. Landowning families typically manage these fields in the main cropping season, but may rent them during the second season, usually based on a 50/50 sharecropping arrangement. Vanilla vines are wound around short, densely-planted support trees. They require hand-fertilization, as natural pollinators do not exist outside the orchid’s home range. Flowering season extends several weeks, but each flower lasts less than a day, demanding several hours of attention each morning. Coffee fields, of the Robusta variety, are typically monoculture, although some are planted among remnant forest trees for shade.

## Data, Farm Family Typology, and Methods

### Socioeconomic Data

This study was conducted in four villages in the foothills adjacent to the Andapa Valley: Mandena, Betsomanga, Befingotra and Andilandrano (Fig. 1). These villages represent the range of agro-ecological conditions and cropping patterns found in the region. For example, Mandena has relatively high population densities, small farms, and a focus on vanilla, while Betsomanga has lower population densities, larger farms, and an emphasis on coffee (Table 1).

**Table 1:** Cropping patterns by village

	Demographics		*Tavy* sector	Paddy sector		Commercial sector	
	Pop. density (pers./km2)	Av. Farm Size (ha) per HH	Average fallow cycle	FAMs cultivate paddy	# seasons	Vanilla:coffee ratio^a^	Av. # CP per HH
Mandena	160	3.3	1:5	80 %	2	4:1	1400
Befinogtra	120	3.5	1:6	30 %	2	2:1	800
Andilandrano	75	7.5	1:8	20 %	2	2:1	1000
Betsomanga	110	5.3	1:7	60 %	1	1:6	1100

All figures refer to 1997 conditions

*HH* Household, *FAM* Family (land management unit), *CP* Commercial Plants (vanilla and coffee)

^a^ratio based on number of plants

Over 300 household interviews were conducted in these villages in 1996–1998 and 2003. Structured questionnaires were used to collect family demographic and production histories, including land ownership, cropping patterns, outputs and farm-gate prices going back to 1987. Most interviews included the husband and wife and took most of a day. Unstructured discussions built a broader understanding of family circumstances. Commodity price information was also collected from processor/exporter business records in the Andapa Valley.

The unit of analysis for these data is the family, which aligns with the land management unit. In order to make multiple-household families comparable to single ones, the data are normalized on a per-household basis. For example, if three households managed nine ha in total, the family is recorded as owning an average of three ha per household, and that figure is used to compare farm sizes across all families. Thus, the 300+ individual households reduce to 155 families.

### Farm Family Typology

**Fig. 3:**
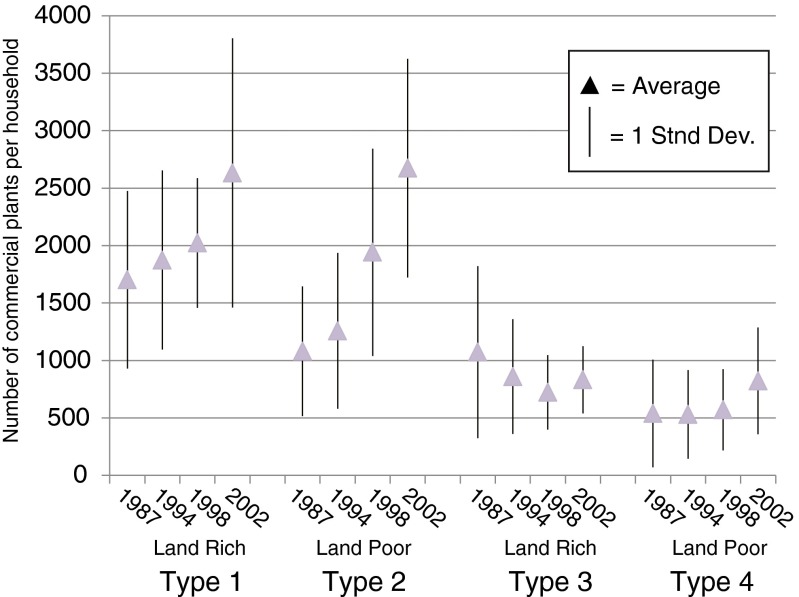
Market engagement by family type

Families are classified into types based on two attributes: farm size and level of commercial production. Land-rich families own ≥5 ha per household; land-poor, <5 ha. Families maintaining at least 1500 commercial plants (coffee and vanilla) per household, or increasing their commercial field sizes by more than 500 plants per household over the 1987 to 1997 time period, engage in high levels of commercial activity (high comm.). Those not meeting either threshold constitute low levels of commercial activity (low comm.). Four farm family types are recognized: Type 1, land rich and high comm.; Type 2, land poor and high comm.; Type 3, land rich and low comm.; and Type 4, land poor and low comm. (Table 2; Fig. 3).

**Table 2:** Farm characteristics by family type

	Demographics					Paddy sector		Commercial sector	
	# FAMs	% FAMs Multi-HH vs Single HH	Av. # Adults per HH ‘87	Av. # New HH’87–‘97	Av. Farm Size (ha, std dev) per HH’87	% FAMs cultivate paddy	^a^Av. % consump. from paddy	Av. # plants per HH 2002	Av. % of land in CP
Type 1	33	36 %	2.2	0.4	9.5 (4.1)	70 %	53 %	2630	10 %
Type 2	27	12 %	1.8	0.1	4.1 (2)	50 %	45 %	2670	40 %
Type 3	42	78 %	2.7	1.4	11.2 (5.1)	45 %	40 %	830	18 %
Type 4	53	30 %	1.8	0.4	3.2 (1.8)	50 %	40 %	820	20 %

Type 1 = Land rich & High comm

Type 2 = Land poor & High comm

Type 3 = Land rich & Low comm

Type 4 = Land poor & Low comm

*HH* Household, *FAM* family (land management unit), *CP* Commercial Plants (vanilla and coffee)

^a^Of paddy cultivators only

### Methods

We assess whether the persistence of self-provisioning in Andapa is consistent with the exogenous or endogenous views using: (1) two simplified conceptual family budget models that compare the profitability of full-market immersion versus self-provisioning; (2) tests of subsistence pressures by family type; (3) on-site observations and other studies in the region; and (4) perception analysis.

#### Family Budget Models

A full-market budget model estimates revenues and expenses for an average-sized household (2 adults, 2 children over and 3 children under the age of 10) enacting full subsistence substitution, purchasing all of its rice with proceeds from its commercial production. We base the parameters of this model on Type 2 families who managed, in 2002, an average of 2600 commercial plants per household with its own labor (Table 2; Fig. 3). This is a conservative figure since some Type 2 units actually managed up to 3500 plants, along with small *tavy* fields (Fig. 3).

A self-provisioning budget model estimates revenues and expenses under a prototypical dual production system. Model parameters assume that the same average-sized household cultivates 1500 commercial plants, 0.8 ha of *tavy*, no paddy, and again relies on its own labor.^1^ This level of commercial production lies at the low end of what Type 1 families typically managed and at the high end of what Type 3 families typically cultivated over the study period (Fig. 3). This level of *tavy* production does not achieve food self-sufficiency. The model household purchased 25–35 % of its subsistence rice in 1987 and 40–50 % in 2007 as *tavy* yields declined.

Model revenues equal the total value from the three crops: vanilla, and coffee and *tavy* (#ha * kg/ha * farm-gate price/kg +/− transportation costs).^2^ Commercial cropping patterns (vanilla:coffee ratios, Table 1) and yields (Table 3) are based on reported averages in each village. *Tavy* yields for 2007 are estimated from a linear projection of each village’s annual average reported yields between 1987 and 2002.

**Table 3:** Average crop yields (kg/ha)

	Vanilla		Coffee		*Tavy* (Avg yield)		
	Avg yield	Low yield	Avg Yield	Low yield	1987	1997	2007^a^
Mandena	1320	825	175 kg/ha	110	975	825	760
Befingotra/Andilandrano	990	500	825 kg/ha	300	825	750	680
Betsomanga	990	165	550 kg/ha	200	775	725	680

^a^ *Tavy* yields for 2007 are estimated from a linear projection of each village’s annual average yields between 1987 and 2002

Vanilla and coffee farm-gate prices are based on green, not processed, commodities. Average prices for the Andapa Region (Fig. 2) account for CAVAGI manipulations and the cut taken by middlemen. Transportation costs, which vary by each village’s proximity to the Andapa Valley, lower those prices by about 1 % in Mandena, 5 % in Befingotra/Andilandrano, and 15 % in Betsomanga.^3^ Transport costs augment the value of rice in each village by these same percentages, based on the assumption that, to replace their cultivated rice, families would purchase it from the Andapa Valley. Input costs are not included in the valuations because farmers do not purchase amendments (e.g., fertilizers) for any of their commodities.

Model expenses include the cost of rice (subsistence needs not fulfilled by their own production), as well as a typical “bundle of goods” that a household purchases to meet its basic needs.^4^ The bundle includes items such as cooking oil and clothes, but omits others such as school fees, medical expenses, and obligations to support family ceremonies. Bundle expenses were available in 1987 and 1997, but not 2007.

Several budget simulations are developed to compare how the two production systems fare under different market and environmental conditions. Budgets under 1987, 1997 and 2007 price conditions avoid vanilla’s extreme upward price swing and capture the overall trends as the price differential between subsistence rice and the commercial crops narrowed over those 20 years (Fig. 2). The 1987 budgets illustrate the impact of the state’s socialist-era price manipulations, while the 2007 budgets represent the combined impact of relatively low commodity prices and high rice prices after market liberalization. Separate budgets for each study village account for village-level variability in vanilla:coffee planting ratios, yields, and farm-gate prices, although Befingotra and Andilandrano were sufficiently similar to aggregate the two villages. Finally, to account for environmental risk, a separate set of “low-yield” budgets was developed for each date and village, assuming that commodity yields were exceptionally low and rice yields remained average. While the low-yield figures are based on recorded occurrences, they did not necessarily occur in the 3 years in question. These budgets are “what-if” scenarios, with 2007 exemplifying a worst-case scenario with both low yields and poor market prices.

#### Subsistence Pressure

We analyze subsistence pressures through demographic stress and food self-sufficiency. Chi-squared tests evaluate the degree of association between commercial-crop expansion and two types of demographic pressures: rapid population growth and new adults. Between 1987 and 1997, nearly all families experienced population growth. Families that added at least three new people over those 10 years constitute rapid growth. A family with a teen turning 16 experienced adult population growth. A family adding at least 200 new plants was considered to be expanding commercial activities.

Food self-sufficiency is defined as meeting minimal subsistence requirements through total rice output (*tavy* plus paddy). The average total production by each family type was divided by the number of consumers per household, normalized to adult equivalents by assuming that children (0–10 years) consume ½ as much, and teens (10–16 years) ¾ as much as adults (see footnote #4). Families producing more than 220 kg of rice per adult equivalent met minimum subsistence.

#### Perception Analysis

Data on family perceptions of their subsistence and commercial activities were generated through an exercise involving 72 of the original 300+ surveyed households across the four study villages, with representation from each family type (Type 1 = 21; Type 2 = 15; Type 3 = 15; Type 4 = 23). An intended Q-method (Brown 1996) was abandoned as some villagers perceived it as a form of witchcraft (*fanafody gasy*; see Evers 2005). A ranking system was devised instead. A head-of-household (male or female, depending on availability) was presented with 28 cards, each with a statement and picture, representing a suite of problems that might make a smallholder unwilling or unable to expand their commercial crops. The research assistant described each card and asked the farmer to decide whether it was a “problem,” “maybe a problem,” or “not a problem,” placing the cards in three piles. The pile of “problem” cards was spread on a table and the farmer selected the two most significant problems (given a rank value of one), and the next three (given a rank value of two). The rest received a rank value of three. The “maybe a problem” and “not a problem” piles received rank values of four and five, respectively.^5^ These data were then aggregated by family type. For example, Type 1’s most important problem was the card that received the lowest average rank value by all Type 1 families (Table 2).

All four family types identified the same eight cards within their top five problems. The study used discriminant analysis, a linear regression technique that allows for more than two categorical dependent variables (Klecka 1980), to determine whether each family type ranked its problems distinctly. In this case, a successful discriminant model is able to classify each family to its type based on how the family ranked the problems. A high rate of misclassification between two family types suggests that farmers from these two types ranked their problems similarly. In this study, a statistically significant 72 % of farmers were correctly assigned to their type, confirming that family types ranked their problems distinctly (based on the maximum chance criterion).

## Results and Discussion

We assess the factors associated with the exogenous and endogenous views in turn. When a factor is relevant to both views, such as risk, we discuss it in detail in only one view.

### Factors Central to the Exogenous View

Do Andapa farmers maintain self-provisioning because the rewards from producing coffee and vanilla are inadequate to support full immersion or because they are constrained from responding to market signals?

#### Transaction Costs

Transactions costs can lower profits from commodities while raising the cost of purchased food, creating a “price band” that can make self-provisioning an economically rational choice (de Janvry *et al.*^1991^; Key *et al.*^2000^; Cadot *et al.*^2010^). The family-budget models reveal the impact of 1) low farm-gate prices set by CAVAGI in the socialist era (1987 in the model); 2) middlemen taking as much as 70 % of the global market price (Cadot *et al*. 2009; 1997 and 2007 in the model), and 3) transportation costs that lower the value of commercial crops, and raise the value of subsistence rice, particularly in outlying villages.

Initial results (Table 4 “Total Crop Value” in 1987 and 1997; 2007 discussed below) indicate that full-market immersion could have produced a higher total crop value than self-provisioning in all villages. For example, in 1987 Mandena, full-market immersion would generate 1,671,000 MGA (Malagasy Ariary) and self-provisioning only 1,046,000 MGA. Additional results (Table 4 “Remaining Revenues”) for these same years reveal that full-market producers could cover the cost of rice purchases and other basic expenses and still be better off than self-provisioners (Table 4).

**Table 4:** Budget comparison under average yield conditions

	Self-provisioning (1000 MGA)			Full-market immersion (1000 MGA)		
	1987	1997	2007	1987	1997	2007
Mandena						
Total crop value^a^	1046	3879	3557	1671	5689	4992
Bundle of goods	(24)	(92)	(n/a)	(24)	(92)	(n/a)
Rice self-provisioned	(59)	(513)	(608)			
Rice purchased	(24)	(342)	(490)	(83)	(855)	(1100)
Remaining revenue^b^	939	2932	2459	1564	4741	3892
Befingotra/Andliandrano						
Total crop value	841	4010	2825	1338	5973	3836
Bundle of goods	(25)	(96)	(n/a)	(25)	96)	(n/a)
Rice self-provisioned	(52)	(485)	(565)			
Rice purchased	(34)	(404)	(578)	(87)	(889)	(1143)
Remaining revenue	730	3025	1682	1227	4988	2692
Betsomanga						
Total crop value	491	3330	1944	685	4367	1888
Bundle of goods	(27)	(105)	(n/a)	(27)	(105)	(n/a)
Rice self-provisioned	(67)	(642)	(774)			
Rice purchased	(28)	(332)	(478)	(95)	(974)	(1252)
Remaining revenue	369	2251	692	563	3288	636

Accounting for transportation costs and production levels at the family labor limit

MGA = Malagasy Ariary; 1 USD = ~1100 MGA in 1987; 5000 MGA in 1997; ~1800 MGA in 2007

^a^Total Crop Value = Revenue from commercial crops + Value of rice self-provisioned

^b^Remaining Revenue = Total Crop Value – Bundle – Value of rice self-provisioned – Rice purchased

The perception analysis produced results somewhat dissonant from those of the budget models. Farm family Types 1 (land rich and high comm.) and 3 (land rich and low comm.) rank “the price is consistently too low” as a significant problem, signaling dissatisfaction with farm-gate prices even though the budget models indicate the prices are not a barrier to commercial expansion. Farmers do not cite middlemen traders or transportation costs as problems (Table 5).

**Table 5:** Farmer perceptions of barriers to commercial expansion

	FAM TYPE & RANK			
	1	2	3	4
Farm size / Land: commercial production:	Rich high	Poor high	Rich low	Poor low
1. Market Incentive				
The price is consistently too low	*		*	
2. Trade and Transportation Transaction Costs				
Buyers who come to my village offer too low a price				
The market is too far away to carry the harvest				
I have no money to hire porters to carry the harvest				
3. Risks (market, environmental and other)				
Prices rise and fall unpredictably				
Rain is not reliable enough				
Storms wipe out my market crops				
Theft is frequent	*			
Neighbors’ cattle damage my market crops				
Neighbors’ fires damage my market crops				
4. Labor				
I have no money to hire workers	**	*	*	*
I reserve my labor for rice production	**	**	**	
There is not enough adult labor in the family		*	*	*
I work for others and have little time left				
I have money, but I cannot find workers				
Workers damage the plants				
My wife is frequently pregnant and cannot work				
I or my wife is frequently sick and cannot work				*
I have too many obligations to my family that take my time				
I have too many obligations to my church or village that take time				
There are other activities that I prefer to do with my time				
5. Land				
I do not own enough land		**		**
My land is of poor quality				
I have family land, but not enough personal land				
I reserve my land for rice production	*	*	**	**
6. Aspirations & Drudgery				
Cultivating market crops is more difficult than rice				
I have the things that want, and do not need more cash				
7. Social leveling				
I fear neighbors will use black magic against me				

** = Highest (1–2) rank

* = Lower (3–5) rank

#### Market Risk

Farmers prioritize safety and reliability when confronting risk to their subsistence security (Lipton 1968; Ortiz 1973; Fafchamps 1992; Scoones 1998; Rosenzweig and Binswanger 1993). Model results for 2007 illustrate the impact of market risk, representing fallen commodities prices and a narrowing of the price gap between rice and the commercial crops (Fig. 2). In that year, “Total Crop Values” and “Remaining Revenues” still indicate superior results for full-market producers in Mandena and Befingotra/Andilarndrano, but not in Betsomanga (Table 4) where high transportation costs and dependence on low-value coffee make full commercialization more vulnerable to low commodity prices.

#### Environmental Risk

All crops in the region experience low yields under adverse environmental conditions, but *tavy* is especially susceptible to weather events and bird infestations. Our study records *tavy* rice losses of 50–75 % in 4 to 5 years out of ten. Vanilla yields, on the other hand, typically drop by 40–50 % once every 5 years, and coffee yields less often. Andapa farmers more commonly rely on commercial revenues, not *tavy*, to ensure their subsistence in such instances. Nevertheless, consistent with dual production systems elsewhere, combined subsistence and commercial production may be viewed as a risk-adverse strategy in the face of potentially low commercial yields (e.g., Ewell and Merrill-Sands 1987; Ellis 2000).

Model results suggest that environmental risk in the commercial sector is not a significant problem. “Remaining Revenues” in the low-yield family-budget models indicate that full-market immersion would be more profitable than self-provisioning in all villages and all years, except Betsomanga in 2007 when commercial revenues (759,000 MGA) would cover only 60 % of the household’s rice needs (1,252,000 MGA) (Table 6). The potential combined impact of low yields, low commercial prices, rising rice prices, and high transportation costs may make self-provisioning a logical choice in this village.

**Table 6:** Budget comparison under low commercial yield conditions

	Self-provisioning (1000 MGA)			Full-market immersion (1000 MGA)		
	1987	1997	2007	1987	1997	2007
Mandena						
Total crop value^a^	676	2618	2452	1044	3557	3121
Bundle of goods	(24)	(92)	(n/a)	(24)	(92)	(n/a)
Rice self-provisioned	(59)	(513)	(608)			
Rice purchased	(24)	(342)	(490)	(83)	(855)	(1100)
Remaining revenue^b^	569	1671	1354	938	2610	2021
Befingotra/Andliandrano						
Total crop value	430	2127	1668	642	2783	1872
Bundle of goods	(25)	(96)	(n/a)	(25)	(96)	(n/a)
Rice self-provisioned	(52)	(485)	(565)			
Rice purchased	(34)	(404)	(578)	(87)	(889)	(1143)
Remaining revenue	319	1142	525	531	1798	729
Betsomanga						
Total crop value	239	1858	1241	281	1983	759
Bundle of goods	(27)	(105)	(n/a)	(27)	(105)	(n/a)
Rice self-provisioned	(67)	(642)	(774)			
Rice purchased	(28)	(332)	(478)	(95)	(974)	(1252)
Remaining revenue	117	779	−11	159	904	−493

Accounting for transportation costs and production levels at the family labor limit

MGA = Malagasy Ariary; 1 USD = ~1800 MGA in 2007

^a^Total Crop Value = Revenue from commercial crops + Value of rice self-provisioned

^b^Remaining Revenue = Total Crop Value – Bundle – Value of rice self-provisioned – Rice purchased

Researchers note that monetary assessments of risk alone, as afforded in our budget-models, do not account for personal and culturally embedded risk judgments and preferences (e.g., Douglas 1985; Tucker *et al.*^2013^). While we did not examine the full realm of risk, perception analysis supports the budget analyses; no family types citied environmental risk as a significant impediment to commercial expansion (Table 5).

#### Labor Constraints and Labor Markets

The family-budget models reveal that family labor is sufficient to support full-market production. Nevertheless, Type 2 (land poor and high comm.) and 3 (land rich and low comm.) families perceive a labor shortage and express the need to hire labor to support such a transition (Table 5).

Cross-farm, but no off-farm, labor markets existed in the villages over the study time frame. About 20–40 % of surveyed households participated in wage labor on other smallholders’ parcels. In most cases, Type 3 and 4 members worked for Type 1 families. No farmers cited an inability to find workers as a problem (Table 4). Nor did they express a concern that hired workers would harm their plants or otherwise not perform adequately (Table 4). Wage laborers did not identify labor constraints regarding their own commercial fields (Table 5).

Wages are not too high either. Using 1997 as an example, the prevailing wage was 5000 MGA/day, or 70,000 MGA for 2 weeks of work. In the vanilla flowering season, one worker can fertilize a field of about 1000 plants, which could yield a conservative figure of 1/3 kg of green pods per plant and generate 2,167,000 MGA. Or, that worker could clear a coffee field of 1000 plants, yielding a conservative ¼ kg of coffee per plant, and generating 1,412,500 MGA.

The perception analysis reveals that family Types 2 and 3 claim to have insufficient household adult labor to expand their commercial fields, but also note that they reserve their own labor for rice production (Table 5). These families effectively admit that subsistence activities create a labor constraint for commercial expansion. Type 1 families, many of whom use hired labor, also note that they reserve family labor for rice. By prioritizing their family labor for rice, these families create the need for cash for hired labor for further commercial expansion.

#### Land Constraints

Commercial crops do not require a lot of space. Even land-poor Type 2 families would only have to convert 5–15 % of their *tavy* land to commercial crops to substitute for self-provisioning. Perception rankings confirm this observation. Land-poor Types 2 and 4 claim that they “do not have enough land” but also declare that they “reserve land for subsistence rice” (Table 5). Their choice to prioritize *tavy* creates the land constraint to commercial expansion. Quality of land is not a factor either. Key informants confirm that commercial crops can be grown nearly anywhere, although they yield better on valley bottoms than on slopes.

Traditional land tenure systems in eastern Madagascar have been shown to impede market engagement, particularly among cultures that root land deep within their belief systems, making it difficult for farmers to consider land as a marketable good (Kistler and Spack 2003). In Andapa it is not uncommon for individuals to sell or rent their private land. The region’s traditional land-tenure system may, however, impose a significant transaction cost on commercial expansion. Family land is reserved for *tavy* and commercial fields can only be planted on private plots. Elders may not be granting enough private plots to facilitate commercial expansion. In fact, the Malagasy state has implemented a land registration program designed to dismantle traditional land tenure systems for that reason (Evers 2005). To test this proposition, we compare the expansion histories of single- and multi-household families. Single-household families control all of their land as private property. Multi-household families only control the land that they have been gifted by the family elder or purchased personally.

Results indicate that multi-household families were much more likely to add both adults and new commercial fields than single ones (Table 7; % FAMs expand CPs), highlighting the strong association between maturing adults and/or new households and commercial expansion. Restricting the analysis to those families who added new adults, multi- and single-household families were equally likely to expand their commercial fields, suggesting that the land-tenure system in question is not a barrier to commercial expansion (Table 7; % FAMs w/ new adults expanded). The perception analysis supports this result. No farmers identified family versus personal land a critical constraint for expanding their commercial fields (Table 5). This interpretation is qualified, however, by the fact that on a per-adult basis single-household families added larger commercial fields than multi-ones (Table 6; Av #CP added per new adult). Yet the larger field sizes may be temporary. Many multi-household families were experiencing such rapid growth (averaging 0.45 new adults per year vs 0.16 among single-household families) that to keep pace they needed to expand their commercial crops at three times the rate of single-household families. This degree of expansion may have needed more time to play out than is captured in our study.

**Table 7:** Influence of family structure and land tenure on market expansion from 1987 to 97

Family structure	# FAMs	Av. FAM Size (ha) per HH^a^	% FAMs added new adults	% FAMs expand CPs^b^	% FAMs w/ new adults expand	Av. #CP added per FAM	Av. # CP added per new adult
Single HH-FAM	96	5.1	54 %	38 %	67 %	1100	800
Multi HH-FAM	65	9.3	80 %	66 %	68 %	975	500

*HH* Household, *FAM* Family (land management unit), *CP* Commercial Plants (vanilla and coffee)

^a^in 1987

^b^CP expansion = 500 or more new plants

In sum, results do not support the exogenous view. With the exception of households in isolated, coffee-dependent Betsomanga, full-market immersion would have been more profitable than maintaining dual production despite the presence of substantial transaction costs and market and environmental risk. Moreover, farmers did not face any of the land, labor, or capital constraints that might stall that transition.

### Factors Central to the Endogenous View

Is a subsistence production logic or a cultural norm held by smallholders themselves making the shift to full-market production a difficult or unwanted transition? We examine several factors relevant to the two endogenous perspectives.

#### Induced Intensification

**Fig. 4:**
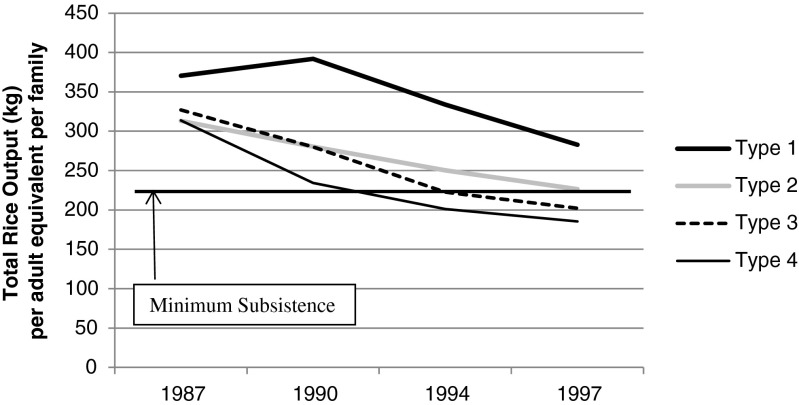
Average total rice output per adult equivalent and minimum subsistence

Andapa farmers may adhere to a subsistence production logic that favors self-provisioning while being pushed into commercial-crop expansion by rising subsistence and land pressures, as documented in other regions of Africa (Pingali *et al.*^1987^; Tiffen and Mortimore 1992).^6^ Subsistence pressures are high in Andapa. Only Type 1 families (land rich and high comm.), which also cultivate the most paddy, maintained food self-sufficiency throughout the 1990s (Fig. 4; Table 2). The other family types began purchasing at least some of their subsistence rice with commercial-crop revenues in the early 1990s, reaching 20–35 % of their rice consumption by 1997. Land pressures are also rising, although commercial expansion is not necessarily linked to these pressures. Chi-square tests reveal statistically significant relationships between commercial-crop expansion and both rapid population growth and adult population growth among the least market-engaged families (Types 3 and 4). No significant relationships are found among the more market-engaged Type 1 and 2 families (Table 8).

**Table 8:** Association between population growth and commercial-crop expansion, 1987–97

	Types 1 and 2				Types 3 and 4			
Rapid population growth		# FAMs No CP Expansion	# FAMs Yes CP Expansion	Total		# FAMs No CP Expansion	# FAMs Yes CP Expansion	Total
	Slow Grw.	9	19	28	Slow Grw.	23	11	34
	Rapid Grw.	4	28	32	Rapid Grw.	24	43	67
	Total	13	47	60	Total	47	54	101
	Pearson’s Chi-Square = 3.395 *p* = 0.65				Pearson’s Chi-Square = 9.2 *p* = 0.002			
Adult population growth		# FAMs No CP Expansion	# FAMs Yes CP Expansion	Total		# FAMs No CP Expansion	# FAMs Yes CP Expansion	Total
	PA. Stable	6	18	24	PA. Stable	20	13	33
	PA Incr.	7	29	36	PA Incr.	27	41	68
	Total	13	47	60	Total	47	54	101
	Pearson’s Chi-Square = 0.26 *p* = 0.61				Pearson’s Chi-Square = 3.9 *p* = 0.048			

*FAM* Family (land management unit), *Rapid Grw.* Families increasing in raw population by 3 or more between 1987 and 1997, *P.A. Incr.* Families increasing in adult population (over 16 years) by 1 or more between 1987 and 1997, *CP* Commercial plants, *Yes CP Expansion* Families increasing number of commercial plants by 200 or more between 1987 and 1997

These results support the interpretation that Type 3 and 4 families (low comm.) are being pushed into commercial expansion, as anticipated by the induced-intensification thesis. However, the production logics typically used to explain smallholders’ resistance to the market (e.g.*,* risk minimization, drudgery of labor, or other aspirations) do not apply in the Andapa case. We treated risk above, concluding that market and environmental conditions do not create insurmountable risks to full-market immersion for most Andapa farmers. Regarding work-loads, subsistence *tavy* actually requires more labor than market crops in terms of both total work hours and toil, especially for weeding. The perception analysis confirms this assessment. Type 3 and 4 farmers did not rank drudgery of labor as an important reason for not expanding their commercial crops (Table 5). Nor did they reveal any other activity competing for labor, such as community, church, or leisure activities (Table 5).

Type 1 families (land rich and high comm.) are not being pushed into the market. They are pursuing the market under relatively low subsistence and demographic pressures. Type 2 families (land poor and high comm.) have been expanding their commercial fields independent of demographic pressure (Table 8) and that shift has undermined their food self-sufficiency (Fig. 4). At the same time, over half of them borrowed and rented land for *tavy* under increasingly unfavorable terms. This suggests that, like Type 1 families, Type 2 families are pursing the market but resisting or held back from full-market immersion, a situation that cannot be explained by the induced-intensification thesis.

#### Cultural Norms and Values

Semi-subsistence communities may resist the capitalist system or seek autonomy from the market to avoid relations of dependency and exploitation (e.g., Wolf 1969; Scott 1985; Ploeg 2008). If Andapa farmers maintain subsistence for such reasons, families that can fully meet their needs through self-provisioning might be expected to engage commercial production the least. Yet Andapa families that produce the most rice per capita (Type 1, Fig. 4) also manage the largest commercial fields (Fig. 3).

Groups may also harbor egalitarian socio-cultural norms and social-leveling mechanisms that discourage individuals from amassing material wealth above a culturally-acceptable level (Wolf 1957; Foster 1965). In southern Madagascar, for example, successful families hold parties (*bilo*) to distribute wealth in exchange for prestige (Fieloux and Lombard 1987). No similar phenomenon occurred or was mentioned during our several extended visits in Andapa.

In other parts of Africa, farmers forego economic opportunities for fear of becoming a target of witchcraft (Platteau 2000). Andapa farmers acknowledge the presence of witchcraft (*fanafody gasy*), but they do not cite it as a problem (Table 5). Conversations with key informants confirmed that the more commercially engaged, wealthy families are not specifically targeted with fanafody gasy*.*^7^ Perhaps this is because socioeconomic stratification is accepted in Tsimihey society. Customary resource ownership, in which land is held at the extended family, household, and individual levels, allows for the accumulation of wealth and prestige (Wilson 1993).

Cultivation practices may be related to socio-religious belief systems, such as maintaining connections to ancestors through land and land uses (e.g., Rappaport 1984), as has been demonstrated in parts of eastern Madagascar (Erdmann 2003). Farmers in Masoala, for example, “root themselves” and establish links to their ancestors (*fombarazana*) through production. *Tavy* may not be essential to this process, however, as at least one family established its fombarazana through paddy and vanilla alone (Keller 2008). The Betsimaraka perform socio-religious practices when preparing land for *tavy* but not paddy. Yet, when these farmers were asked directly why they practice *tavy*, none referred to their belief system, emphasizing instead that they had no alternative (Hume 2006a, b).

The Tsimihety share the tradition of establishing fombarazana through production (Wilson 1993). But Andapa farmers today do not appear to be using *tavy* per se as their means of maintaining spiritual connections. This is evident in the degree to which farmers borrow and rent land for *tavy.* In fact, many farmers actively avoid cultivating *tavy* on their own land if they can secure rental land because renting ensures longer fallows and higher yields on their own land. Rentals last only one season, preventing them from establishing any connections to that land.^8^

Finally, sociocultural norms that ensure food security may impede market expansion when markets undermine that security (Hyden 1980). In some cases, the right to minimum subsistence develops into a broader “moral economy,” with family and village-level networks, institutions, and systems of reciprocity acting as shock absorbers during crises (Scott 1976). Evidence of such a norm can be found in Andapa where all family members inherit land, no family member can be denied access to family land for *tavy*, and family members can request and expect help from richer family members in times of distress. It is not directly apparent, however, that full-market production would threaten this norm. Even in multi-household families, in which such a norm would be expected to have the greatest impact, commercial crops pull relatively little land out of the family land pool. One household’s commercial crop expansion would not detract from another household’s subsistence security. Also, commercial crops actually strengthen a family’s subsistence security as they are less susceptible to environmental risk and generate greater wealth.

Another cultural norm to ensure subsistence security, less noted in the literature, may reside in the social relations of property—a concept articulated by Ferguson (1985, 1990) but never applied to the question of the persistence of self-provisioning. In the Andapa case, rice production, and hence *tavy*, may serve as a form of subsistence security because the social norms dictating how rice can be used are substantially more restrictive than those for “liquid capital” (i.e.*,* cash generated from commercial crops). Beyond basic needs, cash is vulnerable to personal consumption splurges and several culturally-embedded expenditures, such as acts of generosity and hospitality that promote sociality and are believed to engender future prosperity, as well as an acceptance of ‘daring’ (irresponsible) consumption among youth (Walsh 2003). In contrast, rice cannot easily be sold for any reason. Fully substituting *tavy* with commercial-crop revenues would place the entire family budget into a realm of property with social rules that permit or even promote its depletion but few that temper its use.

This explanation is consistent with four observed behaviors in Andapa, First, some individuals spent commercial crop revenues on unwise purchases but never sold their *tavy* for such purchases.^9^ Second, families regularly depleted their commercial crop revenues to meet household needs (e.g., school fees, clothing, medical expenses and funeral ceremonies), but once the money ran out, they never sold *tavy* to meet those needs. They did without. Third, extended family members in distress requested and received cash, but never rice, from wealthier family members. Finally, one family explained that they had no money to take their dying child to a doctor, even though their rice stores were full. Self-provisioning (*tavy*) appears to be a socially acceptable place to render at least some capital illiquid and ensure subsistence security.

This assessment is consistent with other studies in Africa (e.g., Gugerty 2007; Baland *et al.*^2011^) and elsewhere (e.g., Banergee and Duflo 2011) that illustrate a variety of strategies used to maintain some assets in parts of a portfolio that protect them from personal consumption splurges or from the extended family and community demands. 10 This reserve ensures, at most, minimum subsistence. It does not facilitate the accumulation of wealth. Importantly, this norm could explain why Type 1 and 2 families (high comm.) resist full immersion into the market, but it cannot explain why Type 3 and 4 families (low comm.) produce commercial crops below their capacity.

## Conclusions and Implications

Our results do not support the exogenous thesis of why Andapa farmers maintained self-provisioning over the period of study. Despite market imperfections and risk, full-market immersion appears to have been more profitable than dual production, except in the case of coffee-dependent farmers in Betsomanga. Nor do farmers face land or labor constraints, even in the most marginalized cases.

Our results do support an endogenous interpretation, but none of the explanations typically associated with this thesis prove relevant to the Andapa case. Subsistence pressures appear to be pushing Type 3 and 4 farm families (low comm.) into the market, as anticipated by induced-intensification, but the thesis fails to account for levels of production that are far below apparent capacity. Nor does induced intensification explain why Type 1 and 2 families (high comm.) clearly pursue the market yet resist full immersion.

A stronger case can be made for the view that a cultural norm residing in subsistence security is impeding them (at least Type 1 and 2 families) from full-market immersion. Yet, the typical assumption embedded in this view—that commercial crops compete with and undermine more reliable and accessible subsistence crops—does not resonate in Andapa. Instead, this study links adherence to self-provisioning to an institution that has not yet been applied to this question – the social relations of property. While the evidence is not definitive, if this interpretation is correct, self-provisioning in Andapa produces a secure asset to deposit in a socially-sanctioned banking system, withdrawn by consumption of the farm family alone.

Further research is needed to fully develop and verify this hypothesis. If correct, the implications are significant for Malagasy development and environmental policy. To date, policymakers have focused on encouraging commercial expansion through market liberalization. Environmental NGOs expected this transition to launch subsistence substitution. Farmers would replace *tavy* with vanilla and coffee in pursuit of higher market rewards and in turn reduce land pressure on the nearby nature reserves (WWF-Madagascar 2012). It hasn't worked. Liberalization has spurred commercial production but fallen short of supplanting self-provisioning.

We hypothesize that this failure stems in part from the fact that liberalization policies do not address the social relations of property as they affect subsistence security. More recently, policymakers have been targeting the lack of credit and savings institutions in the region, but appear to misread the actual role that those institutions may need to play (e.g., IFAD 2012). Policy documents highlight the need for savings cooperatives to provide a safety net in low-price years and reduce unwise consumption splurges (IFAD 2012). The first reason is appropriate, although in fewer cases than may be appreciated, given the results reported here. The second reason only partially addresses pressures on cash, which runs deeper than personal splurges. If savings accounts are to replace the protective role that *tavy* plays they must be culturally sanctioned in the way that *tavy* holds subsistence rice. Strengthening the social rules concerning cash may also have negative tradeoffs, possibly undermining the important role that cash plays in alleviating subsistence risk at the extended family level. A single savings account, within a single property domain, may not be able to uphold the very different roles that the two traditional property domains, *tavy* and cash, currently play.

The persistence of swidden across the tropical world continues to attract interest, with many studies highlighting conditions similar to those in Andapa, where land pressures and economic conditions favor more intensive and commercial cultivation (e.g., Sulistyawati *et al*. 2005; Mertz *et al*. 2013; Van Vliet *et al.*^2013^). Few, if any, address endogenous factors before concluding that exogenous constraints are inhibiting full-market immersion -- especially among more marginalized households (e.g., Schmook *et al.*^2013^). Our research suggests that endogenous factors, including the social relations of property, need to be considered.

## Footnotes

1 Approximately 60 % of families across all study villages relied solely on *tavy* (no paddy) for rice production.

2 Farmers count plants, not hectares. Field size conversions are based on 3300 vanilla plants and 1100 coffee plants per ha.

3 Farmers typically carry their own crops to market, but transportation costs were considered since injuries or other circumstances can force them to incur these costs.

4 Based on a socioeconomic survey conducted by WWF-Andapa in 1995. An average-sized family (see text) consumes 1100 kg of rice per year (adults: 220 kg; children <10 year: 110 kg; teen 10–16 years: 165 kg.). The ‘bundle’ includes: salt, sugar, oil, kerosene, soap, blankets, and clothes.

5 If they chose fewer than five “problem” cards, they had to identify their two greatest problems and then decide how many cards to give rank two and three status. If they chose more than eight “problems” cards, the ninth and above fell in the fourth rank.

6 Much of this work describes a transition in which population pressures induce the intensification of subsistence production first, and only later drive commercial cropping. In some cases, both subsistence and commercial crops are maintained.

7 Informants explain that most villagers attend church and do not practice fanafody gasy. Those who do typically use it to cure sicknesses, prepare for a new house or a marriage, or encourage events to go as they would prefer.

8 The impact of borrowing and renting land on socio-religious practices has not been explicitly investigated (Bloch 1971). Actions that establish *tanindrazana* (land of the ancestors), including childbirth and the reburial of ancestral bones on that land (Keller 2008), imply, however, that they occur on their own, not rented, land.

9 Brown (2009) describes spending splurges in neighboring Antalaha on items such as stereo systems and expensive tennis shoes during the 2003/4 spike in vanilla prices, making them vulnerable to the price crash in 2005.

10 Baland *et al.* (2011) describes farmers who hide their own cash and take loans from banks (paying costly interest) because it is easier for them to refuse demands from family members on their borrowed cash than to protect their own cash from those demands. Gugerty (2007) describes ROCAs in Africa (Rotating Savings and Credit Associations where individuals agree to a schedule of periodic payments in return for a lump-sum payment at a future date) that exist even where formal banking institutions are available because ROCAs are more acceptable than banks as a place to render capital illiquid.
